# Development and Validation of a Real-Time PCR Assay for Rapid Detection of Candida auris from Surveillance Samples

**DOI:** 10.1128/JCM.01223-17

**Published:** 2018-01-24

**Authors:** L. Leach, Y. Zhu, S. Chaturvedi

**Affiliations:** a Mycology Laboratory, Wadsworth Center, New York State Department of Health, Albany, New York, USA; b Department of Biomedical Sciences, School of Public Health, University at Albany, Albany, New York, USA

**Keywords:** Candida auris, surveillance samples, TaqMan chemistry, assay validation, real-time PCR assay

## Abstract

Candida auris is an emerging multidrug-resistant yeast causing invasive health care-associated infection with high mortality worldwide. Rapid identification of C. auris is of primary importance for the implementation of public health measures to control the spread of infection. To achieve these goals, we developed and validated a TaqMan-based real-time PCR assay targeting the internal transcribed spacer 2 (*ITS*2) region of the ribosomal gene. The assay was highly specific, reproducible, and sensitive, with the detection limit of 1 C. auris CFU/PCR. The performance of the C. auris real-time PCR assay was evaluated by using 623 surveillance samples, including 365 patient swabs and 258 environmental sponges. Real-time PCR yielded positive results from 49 swab and 58 sponge samples, with 89% and 100% clinical sensitivity with regard to their respective culture-positive results. The real-time PCR also detected C. auris DNA from 1% and 12% of swab and sponge samples with culture-negative results, indicating the presence of dead or culture-impaired C. auris. The real-time PCR yielded results within 4 h of sample processing, compared to 4 to 14 days for culture, reducing turnaround time significantly. The new real-time PCR assay allows for accurate and rapid screening of C. auris and can increase effective control and prevention of this emerging multidrug-resistant fungal pathogen in health care facilities.

## INTRODUCTION

Candida auris is emerging as a multidrug-resistant yeast causing invasive health care-associated infection with high mortality worldwide (1–6). Recently, it has emerged in the United States, with the majority of cases reported from New York (https://www.cdc.gov/fungal/diseases/candidiasis/candida-auris.html). The precise mechanisms leading to the emergence and spread of C. auris are currently unclear, but it raises several serious concerns for public health. Many isolates are multidrug resistant (MDR), with some isolates having elevated MICs to all three classes of antifungals (7–11).

Many commercially available biochemically based tests, including analytical profile index (API) strips and Vitek 2, misidentify C. auris as Candida haemulonii, Candida famata, and/or Rhodotorula glutinis. Matrix-assisted laser desorption ionization–time of flight mass spectrometry (MALDI-TOF MS) has emerged as a successful platform for the accurate identification of yeasts, including C. auris (10, 12, 13). Molecular methods based on sequencing of the internal transcribed spacer (ITS) and D1/D2 regions of the ribosomal gene can successfully differentiate C. auris from other closely related species (1). Although these techniques are excellent for the identification of C. auris, they are time-consuming, as they require an isolate. Recently, a real-time PCR assay using melt curve analysis to distinguish C. auris from other closely related species was reported (14).

Here we report the development and validation of a TaqMan chemistry-based real-time PCR assay targeting the *ITS2* gene of C. auris. Our results indicate that the real-time PCR assay is an important tool for rapid detection of C. auris from surveillance samples and able to provide more efficient control and prevention of the spread of this emerging multidrug-resistant yeast in health care facilities.

## MATERIALS AND METHODS

### C. auris real-time PCR assay.

The multiple alignment of the *ITS2* genes from all four clades of C. auris and closely related pathogenic yeasts revealed a region highly specific for C. auris (see Fig. S1 in the supplemental material). Primers and a probe were designed from this region using the PrimerQuest program (Integrated DNA Technologies, Coralville, IA). Primers and a probe were also designed for an inhibition control from a *bicoid* gene. Sequences for primers and probes for C. auris ITS2 gene were as follows: V2424F (CAURF), 5′-CAGACGTGAATCATCGAATCT-3′; V2425P (CAURP), 5′-/56-carboxyfluorescein (FAM)/AATCTTCGC/ZEN/GGTGGCGTTGCATTCA/3IABkFQ/-3′; and V2426 (CAURR), 5′-TTTCGTGCAAGCTGTAATTT-3′. Those for the *bicoid* gene were as follows: V2375 (BICF), 5′-CAGCTTGCAGACTCTTAG-3′; V2384 (BICP), 5′/Cy3/AACGCTTTGACTCCGTCACCCA/3IAbRQSp/-3′; and V2376 (BICR), 5′-GAATGACTCGCTGTAGTG-3′. All primers and probes were obtained from Integrated DNA Technologies.

Each sample was tested in duplicate in 20-μl volumes using an optical 96-well reaction plate. Each reaction mixture contained 1× PerfeCTa Multiplex qPCR ToughMix (Quanta Biosciences), a 500 nM concentration of each C. auris primer (V2424 and V2426), a 100 nM concentration of each *bicoid* primer (V2375 and V2376), a 100 nM concentration each of C. auris (V2425P) and *bicoid* probe (V2384P), and 5 μl of DNA extracted either from C. auris or from the surveillance samples. Each PCR run also included 5 μl of positive extraction (C. auris M5658; 10^3^ CFU/50 μl) and positive amplification (C. auris M5658; 0.02 pg/μl) controls, as well as 5 μl of negative extraction (reagents only) and negative amplification (sterilized nuclease-free water) controls. To prevent any cross-contamination, a unidirectional workflow was followed by keeping reagent preparation, specimen preparation, and amplification/detection areas separate.

Cycling conditions on the ABI 7500 FAST were 95°C for 20 s, followed by 45 cycles of 95°C for 3 s and 60°C for 30 s. Based on receiver operating characteristic (ROC) curve analysis, a cycle threshold (*C_T_*) value of ≤37 was reported as positive and >37 was reported as negative for swabs. Similarly, a *C_T_* value of ≤38 was reported as positive and >38 was reported as negative for sponges. Specimens were reported as inconclusive if PCR inhibition was observed for either swabs or sponges.

### Analytical sensitivity, reproducibility, and specificity.

C. auris isolate M5658, a major genotype belonging to the South Asia clade and involved in the current outbreak in New York, was used to assess the analytical sensitivity of the real-time PCR assay. In brief, C. auris was grown overnight on a Sabouraud dextrose agar (SDA) plate, and colonies were then scraped and suspended in phosphate-buffered saline (PBS) containing 0.01% bovine serum albumin (BSA). Cells were counted with a hemocytometer, serial dilutions were prepared, and the cell suspension was plated to obtain CFU. The yeast cell suspension was adjusted based on CFU recovered, and 50 μl of each cell dilution containing 2 beads (3 mm; Fisher Scientific) was processed for DNA extraction by first freezing cells at −20°C for 30 min, followed by heating at 70°C for 30 min and then bead beating at 4,700 rpm for 15 s in a Precellys 24 homogenizer (Bertin Technologies, France). Five microliters of extracted DNA from each cell dilution was tested in a real-time PCR assay in duplicate. The analytical sensitivity of the real-time PCR assay was determined by performing three independent DNA extractions. The analytical reproducibility of the real-time PCR assay was determined by extracting DNA from C. auris at a high (10^5^ CFU/50 μl), moderate (10^3^ CFU/50 μl), or low (10^2^ CFU/50 μl) concentration, and 5 μl of extracted DNA was run in triplicate on three different days (interassay), as well as within the same day (intra-assay) of testing. The analytical specificity of the real-time PCR assay was determined by testing approximately 1 ng of genomic DNA (gDNA) from a panel of reference and clinical isolates of fungi (yeasts and molds), bacteria, parasites, and viruses.

The analytical reproducibility and specificity of the real-time PCR assay were also assessed in previously determined negative surveillance swab and sponge matrices. In brief, 200 μl of liquid from swabs or sponges were pooled separately. Each pooled swab or sponge liquid was washed by centrifugation, resuspended in PBS-BSA to the original volume used for pooling, and then aliquoted into 43 μl. Every 10 aliquots (swab or sponge) were spiked randomly with 5 μl of a high (10^5^ CFU/50 μl), moderate (10^3^ CFU/50 μl), or low (10^2^ CFU/50 μl) concentration of C. auris and 2 μl of *bicoid* DNA (5 × 10^−3^ ng/μl). Ten swab and sponge aliquots were also spiked with moderate numbers (10^3^/50 μl) of other Candida spp. and *bicoid* DNA. All spiked samples were lysed and centrifuged, and 5 μl of supernatant was directly tested in duplicate using the C. auris real-time PCR assay.

### C. auris real-time PCR assay surveillance sample study.

To evaluate the assay for identification of C. auris directly from surveillance samples, 623 surveillance samples, including 365 clinical swabs (axilla-groin, axilla, groin, nares, ear, rectal, and wound) and 258 environmental sponges from various health care facilities, were tested. Details of sample processing and DNA extraction are provided below.

Each sponge-stick (3M Health Care, St. Paul, MN) received after environmental swabbing of various facilities was placed in a bag containing 45 ml of PBS with 0.02% Tween 80 (PBS-T80). Each bag was gently shaken for 1 min at 260 rpm in a Stomacher 400 circulator (Laboratory Supply Network, Inc., Atkinson, NH). The suspension from the bag was transferred into a 50-ml conical tube and centrifuged at 4,000 rpm for 5 min. The supernatant was decanted, leaving approximately 3 ml of liquid in the bottom of the tube. The concentrated sponge liquid was transferred into 3 separate sterilized tubes (Sarstedt Inc., Newton, NC). After processing of a specific volume (described below) for PCR and culture, the remaining liquid was saved at 4°C.

Patient swabs that were received in liquid transport medium (Becton & Dickinson Company, Franklin Lakes, NJ) were vortexed vigorously for 30 s, the swab was removed, and approximately 1 ml of liquid was transferred into a 2-ml sterilized tube (Sarstedt Inc.). The patient swabs that were received in solid transport medium (BD CultureSwab, Becton & Dickinson) were first removed from the solid medium and then placed in a glass tube containing 1 ml of PBS-T80. After vortexing for 30 s in the vortexer, the swab was removed and liquid was saved in a 2-ml sterilized tube. After processing of a specific volume (described below) for PCR and culture, the remaining liquid was saved at 4°C.

For DNA extraction, 1 ml of sponge liquid and 200 μl of swab liquid were centrifuged at 13,000 rpm for 5 min. The recovered pellet was washed twice with PBS-BSA and resuspended in 48 μl of PBS-BSA. Two glass beads and 2 μl of *bicoid* inhibition control plasmid DNA (5 × 10^−3^ ng/μl) were added to each tube, followed by DNA extraction as described above for C. auris cell suspension, and 5 μl of extracted DNA was used in the real-time PCR assay in duplicate. The PCR results were then compared with culture results prospectively.

### Statistical analysis.

GraphPad Prism 5 software for Mac (GraphPad Software, Inc., La Jolla, CA) was used for statistical analysis. The Student *t* test was used for analysis of the means, and a *P* value of <0.05 was considered statistically significant. To assess the diagnostic value of the real-time PCR assay in C. auris detection from surveillance samples, the culture method was selected as the “gold standard.” According to results obtained from both methods, a receiver operating characteristic (ROC) curve (MedCalc software version 17.8; Ostend, Belgium) analysis was generated, with the area under the curve (AUC) of real-time PCR calculated. The optimal diagnostic cutoff value was determined by calculating the Youden index of the ROC curve. Statistical significance was set as a *P* value of <0.05.

## RESULTS

### Assay sensitivity, specificity, and reproducibility.

**FIG 1:**
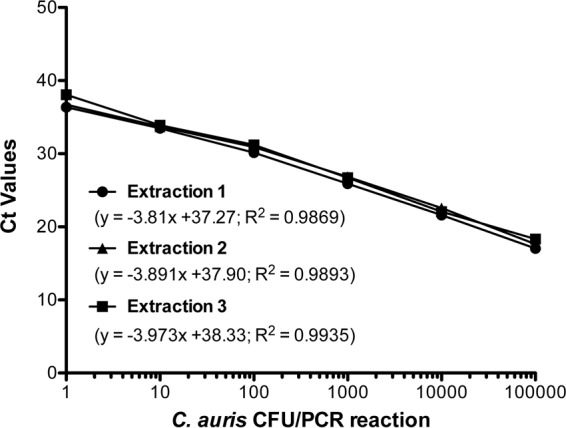
Real-time PCR assay sensitivity. Candida auris cell dilution series containing 10^−1^ to 10^5^ CFU/PCR reaction were run in duplicate on three different days. The slopes and correlation coefficients (*R*^2^) are shown. The detection limit of the assay was 1 C. auris CFU/PCR reaction.

The C. auris real-time PCR assay was linear over 5 orders of magnitude, and the limit of detection of the assay was 1 C. auris CFU/PCR using 45 PCR cycles in all three extraction processes, confirming the high analytical sensitivity of the assay (Fig. 1). The assay was highly reproducible, as it produced consistent *C_T_* values for a given cell concentration on three different days of testing as well as within the same day of testing. The coefficient of variance (CV) was less than 5%, confirming the high reproducibility of the assay (see Tables S1 and S2 in the supplemental material). Next, we determined the assay specificity by using genomic DNA (gDNA) from closely and distantly related fungal, bacterial, parasitic, and viral pathogens. The real-time PCR assay was highly specific, as none of the other organisms cross-reacted, while C. auris organisms belonging to all known phylogenetic clades by whole-genome sequencing yielded positive results (see Table S3 in the supplemental material).

The assay reproducibility and specificity were also evaluated in swab and sponge matrices by spiking 40 swabs and 40 sponge samples with either C. auris or other Candida spp. in a blinded fashion. All the swab and sponge samples spiked with low to high numbers of C. auris were positive by real-time PCR assay, with a CV of less than 5%. However, one set of sponge samples spiked with a high number of C. auris cells revealed a marginally high CV, 5.71%. All swab and sponge samples spiked with other Candida spp. were negative (see Table S4 in the supplemental material).

### Performance of C. auris real-time PCR assay on surveillance samples.

A total of 365 patient swabs and 258 environmental sponges were tested concurrently by culture and real-time PCR. These investigations were done in a blinded fashion, and then results were matched to determine the performance of the real-time PCR assay. Among the 365 patient swabs tested, 46 were true positive and 310 were true negative. Six of the culture-positive swabs were negative by the real-time PCR assay (false negative), while the assay picked up C. auris DNA from 3 culture-negative swab samples (false positive). Further analysis indicated that five of the six culture-positive swabs harbored fewer than 10 yeast cells in the 50 μl of concentrated swab suspension used for DNA extraction and hence fell below the detection limit of the real-time PCR assay (1 C. auris CFU/5 μl), and one swab had a PCR-inhibitory substance. Using the culture method as the gold standard, the accuracy of the real-time PCR assay for the detection of C. auris from patient swabs was determined to be 98%, with a clinical sensitivity and specificity of 89% and 99%, respectively (Table 1).

**TABLE 1:** Comparison of culture and real-time PCR results of swab surveillance samples^*a*^

Real-time PCR result	No. of swabs with indicated culture result		Accuracy (%)	Sensitivity (95% CI)	Specificity (95% CI)	PPV (%)	NPV (%)
	Positive	Negative					
Positive	46	3	98	89	99	94	98
Negative	6	310		(77–96)	(97–100)		

a CI, confidence interval; PPV, positive predictive value; NPV, negative predictive value.

Real-time PCR analysis of 258 sponge samples revealed 32 true positive and 200 true negative (Table 2). The real-time PCR assay detected C. auris DNA from an additional 26 culture-negative sponge samples (false positive). Again, based on culture as the gold standard, the accuracy of the real-time PCR assay for detection of C. auris from sponge samples was 90%, with a clinical sensitivity and specificity of 100% and 89%, respectively (Table 2).

**TABLE 2:** Comparison of culture and real-time PCR results of sponge surveillance samples

Real-time PCR result	No. of sponges with indicated culture result		Accuracy (%)	Sensitivity (95% CI)	Specificity (95% CI)	PPV (%)	NPV (%)
	Positive	Negative					
Positive	32	26	90	100	89	55	100
Negative	0	200		(89–100)	(84–92)		

**FIG 2:**
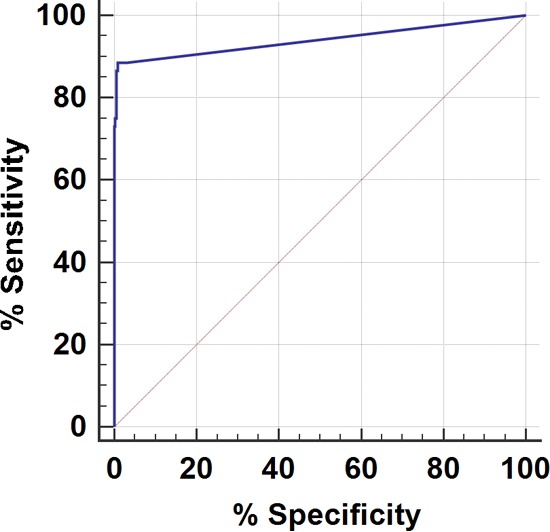
ROC curve analysis of C. auris real-time PCR assay for patient (swab) surveillance samples. A ROC curve was plotted by calculating the sensitivity and specificity of the real-time PCR *C_T_* values compared to culture results for swabs.

**FIG 3:**
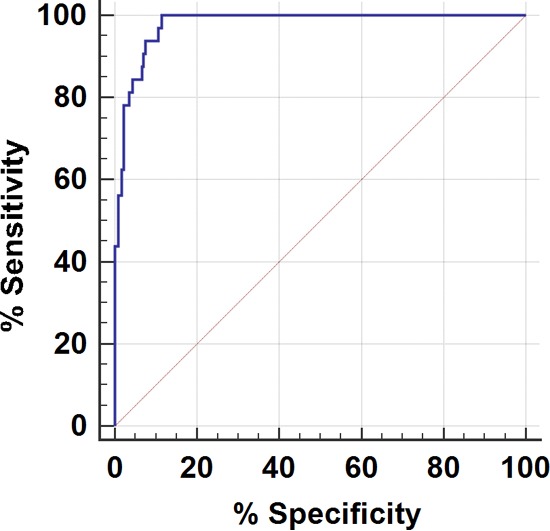
ROC curve analysis of C. auris real-time PCR assay for environmental (sponge) surveillance samples. A ROC curve was plotted by calculating the sensitivity and specificity of the real-time PCR *C_T_* values compared to culture results for sponges.

We also used ROC curve analysis as a statistical tool for the diagnostic evaluation of the real-time PCR assay on surveillance samples. Compared with the culture method, the areas under the ROC curves for the real-time PCR assay were 0.940 for swabs and 0.978 for sponges. Both values were statistically significant (*P* < 0.0001) compared with the value of 0.5, which corresponds to the chance with no diagnostic value (Fig. 2 and 3). These results demonstrated that the real-time PCR assay has a highly accurate rate of detection of C. auris from the surveillance samples. The calculated Youden index for the ROC curves reached maximum *C_T_* cutoff values of ≤37.0 and ≤38.0 for swabs and sponges, respectively, which can be used as the optimal diagnostic cutoff values for the surveillance samples.

## DISCUSSION

**FIG 4:**
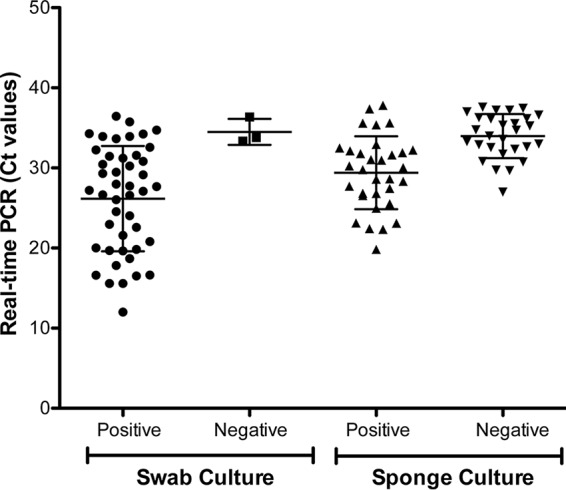
C. auris DNA detection in swab and sponge samples by real-time PCR assay. Crude DNA from surveillance samples was extracted by the bead-beating method, and 5 μl of extracted DNA was subjected to the real-time PCR assay. Results are shown as a scatter plot based on *C_T_* counts for surveillance samples either positive or negative by culture. Error bars indicate SDs. The mean *C_T_* values between culture-positive and culture-negative samples for swabs and sponges were statistically significant (*P* < 0.05).

Candida auris has emerged as a multidrug-resistant yeast causing substantial mortality in health care settings worldwide. Rapid identification of C. auris directly from patient samples is of primary importance for the administration of empirical antifungal therapy and for the implementation of public health measures to control the spread of infection. In this investigation, we developed a real-time PCR assay for rapid detection of C. auris from surveillance samples. The real-time PCR assay provided rapid results within 4 h of sample processing, compared to the much slower culture results, which may take anywhere from 4 to 14 days. The assay was highly sensitive, with a detection limit of 1 C. auris CFU/PCR. The high sensitivity of the real-time PCR assay is not surprising since the multicopy *ITS2* was used as a gene target. More importantly, the assay was highly specific, as no cross-reactivity was observed with all known closely and distantly related yeasts and other pathogens. Also, surveillance samples that were negative for C. auris DNA harbored other organisms, which did not cross-react, further confirming the high specificity of the real-time PCR assay (see Fig. S2 in the supplemental material). For culture-positive surveillance samples, the sensitivities of the real-time PCR assay for swabs and sponges were 89% and 100%, respectively. The reason for variability in sensitivity observed between swabs and sponges is not clear. One possibility could be the use of a larger volume of sponge (1 ml) versus swab (0.2 ml) resulting in the presence of more C. auris yeasts prior to DNA extraction and real-time PCR assay. Among the culture-negative surveillance samples, 26 sponge samples (12%) were positive by PCR and only 3 swab samples (1%) were positive by PCR, resulting in clinical specificities of 89% and 99% for sponge and swab, respectively. A scatter plot (Fig. 4) revealed a wider range of *C_T_* values for culture-negative sponges than for culture-negative swabs, indicating that C. auris present in the environmental surfaces was either dead or growth defective. A recent study reported that C. auris can survive up to 2 weeks on plastic surfaces such as those found in health care settings (15). It is also important to note that environmental surfaces in health care facilities are consistently being cleaned with a variety of disinfectants, some of which are less potent for specific organisms than others. As a result, the real-time PCR assay possibly picked either residual DNA or dead or culture-defective C. auris. It is well known that bacteria and yeasts require quorum sensing to grow and multiply (16), and the absence of C. auris recovery in cultures from a PCR-positive patient or environmental site might reflect a failure in this system. Since PCR can pick up C. auris even when culture results are negative, it can provide increased insight into possible modes of dissemination within health care facilities.

Our real-time PCR assay is able to detect all known clades of C. auris as reported by whole-genome sequencing (4), indicating that it has the potential for broader applications worldwide. Recently, Kordalewska et al. (14) described a real-time PCR assay utilizing a probe targeting the *ITS1* and *ITS2* region of the ribosomal gene followed by melt curve analysis to distinguish C. auris from other closely related Candida spp. However, the distinguishing features of our assay include the use of TaqMan probe chemistry, higher sensitivity (with a limit of detection of 1 C. auris CFU/PCR), inclusion of all known clades of C. auris as reported by whole-genome sequencing, and direct utilization of the test for detection of C. auris from large numbers of surveillance samples. Our assay provides a new and superior method to increase accurate and rapid screening of C. auris. Improvements in accuracy and speed of detection of C. auris from surveillance samples will help in devising strategies for containment of infected patients or colonized individuals and promote prompt cleaning of environmental surfaces so a further spread of C. auris can be prevented.

In summary, C. auris is a well-known nosocomial global pathogen that has recently emerged as a significant threat in the United States, with the number of reported clinical cases identified by culture increasing rapidly, from 7 in August 2016 to 77 in May 2017 and 98 in mid-July 2017 (17). In the face of this critical development, our newly developed real-time PCR assay delivers badly needed, highly accurate, and rapid screening of C. auris from surveillance samples, promising more effective control to prevent the spread of this emerging multidrug-resistant fungal pathogen in health care facilities (2–4, 7).

## Supplementary Material

Supplemental material
