# Supplementary material for: Development and Validation of a Real-Time PCR Assay for Rapid Detection of Candida auris from Surveillance Samples
Source: J Clin Microbiol. 2018 Jan 24;56(2):e01223-17. doi: 10.1128/JCM.01223-17 (PMC5786737; doi:10.1128/JCM.01223-17)
Supplement: Supplemental material [file supp_56_2_e01223-17__index.html]

Supplemental material 

# Development and Validation of a Real-Time PCR Assay for Rapid Detection of Candida auris from Surveillance Samples

## Supplemental material

- Supplemental file 1 -

  Legends to Fig. S1 and S2; Tables S1 (Interassay reproducibility of real-time PCR assay), S2 (Intra-assay reproducibility of real-time PCR assay), S3 (*C. auris* real-time PCR assay specificity), and S4 (*C. auris* detection in spiked surveillance samples by real-time PCR assay); and Fig. S1 (Multiple alignment of *ITS2* gene of *C. auris* and other closely related *Candida* species) and S2 (Specificity assessment of *C. auris* real-time PCR assay in actual surveillance samples negative for *C. auris* DNA)

  PDF, 681K
- Supplemental file 2 -

  Fig. S1 (Multiple alignment of *ITS2* gene of *C. auris* and other closely related *Candida* species)

  PDF, 1.8M
- Supplemental file 3 -

  Fig. S2 (Specificity assessment of *C. auris* real-time PCR assay in actual surveillance samples negative for *C. auris* DNA)

  PDF, 709K
