# Supplementary material for: Development and Validation of a Real-Time PCR Assay for Rapid Detection of Candida auris from Surveillance Samples
Source: J Clin Microbiol. 2018 Jan 24;56(2):e01223-17. doi: 10.1128/JCM.01223-17 (PMC5786737; doi:10.1128/JCM.01223-17)
Supplement: Supplemental material [file JCM.01223-17_zjm999095818s1.pdf]

JCM01223-17R1

Supplementary Figure 1. Design of primers and probe from *ITS2* gene. Multiple alignments of *ITS2* gene comprising all phylogenetic clades of *C. auris* and other closely related yeast species resulted in a combination of sequences used for the design of primers and probe for *C. auris.* The GenBank numbers for strains used for alignments are AB375772 *Candida auris* East Asia clade; KC692039 *Candida auris* South Asia clade; KJ126758 *Candida auris* Africa clade; KT305985 *Candida auris* South America clade; KX810325 *Candida auris* East Asia clade; KX870921 *Candida auris* South Asia clade; KX870919 *Candida duobushaemulonii*; KX870918 *Candida haemulonii*; JX459678 *Candida pseudohaemulonii*; AF246989 *Candida krusei*; KU729100 *Candida lusitaniae*.

Supplementary Figure 2. Specificity assessment of *C. auris* real-time PCR assay in actual surveillance samples negative for *C. auris* DNA. The surveillance samples harboring other organisms (recovered in culture) or harboring no organisms (no growth in culture) did not cross react in *C. auris* real-time PCR assay further confirming high specificity of the real-time PCR assay. The organisms recovered are listed below the pie chart for swabs (A) and sponges (B).

Supplementary table 1. Inter-assay reproducibility of the real-time PCR assay

**Level**	**Yeast CFU/ 50 µl**	**Yeast CFU/ PCR Rxn**	**Day 1**			**Day 2**			**Day 3**			**Mean Ct ± SD**	**% CV**
			**Ct 1**	**Ct 2**	**Ct 3**	**Ct 1**	**Ct 2**	**Ct 3**	**Ct 1**	**Ct 2**	**Ct 3**		
High	10^5	10^4	21.99	21.62	21.61	22.56	22.59	22.83	22.02	22.14	22.15	22.17 ± 0.42	1.90
Moderate	10^3	10^2	30.14	30.15	30.15	30.84	30.96	30.40	31.66	30.77	31.01	30.68 ± 0.51	1.67
Low	10^2	10^1	33.30	33.65	33.28	33.66	33.76	33.80	33.91	33.86	34.30	33.72 ± 0.31	0.92

Supplementary table 2. Intra-assay reproducibility of the real-time PCR assay

**Level**	**Yeast CFU/50 µL**	**Yeast CFU/Rxn**	**Ct 1**	**Ct 2**	**Ct 3**	**Mean Ct ± SD**	**% CV**
High	10^5	10^4	21.99	21.62	21.61	21.74 ±0.22	0.99
Moderate	10^3	10^2	30.14	30.15	30.15	30.15 ±0.01	0.02
Low	10^2	10^1	33.30	33.65	33.28	33.41 ±0.21	0.62

Supplementary Table 3. *C. auris* real-time PCR assay specificity

Isolate No.	Organism	Source	*C. auris* Mean Ct
-	NTC	-	0
Candida auris			
M5658	*C. auris* (South Asia, Clade I)	New York, USA	18.78
M5587	*C. auris* (South Asia, Clade I)	New York, USA	18.46
M5695	*C. auris* (South Asia, Clade I)	New York, USA	18.39
M5676	*C. auris* (South Asia, Clade I)	New York, USA	18.72
AR0382	*C. auris* (South Asia, Clade I)	FDA-CDC AR Bank	18.39
AR0387	*C. auris* (South Asia, Clade I)	FDA-CDC AR Bank	18.81
AR0388	*C. auris* (South Asia, Clade I)	FDA-CDC AR Bank	17.85
AR0389	*C. auris* (South Asia, Clade I)	FDA-CDC AR Bank	18.14
AR0390	*C. auris* (South Asia, Clade I)	FDA-CDC AR	18.36
M5692	*C. auris* (East Asia, Clade II)	New York, USA	19.06
M5657	*C. auris* (East Asia, Clade II)	New York, USA	18.63
M5691	*C. auris (*East Asia, Clade II)	New York, USA	18.80
AR0381	*C. auris (*East Asia, Clade II)	FDA-CDC AR Bank	17.68
AR0383	*C. auris* (Africa, Clade III)	FDA-CDC AR Bank	18.63
AR0384	*C. auris* (Africa, Clade III)	FDA-CDC AR Bank	19.16
AR0385	*C. auris* (South America, Clade IV)	FDA-CDC AR Bank	18.79
AR0386	*C. auris* (South America, Clade IV)	FDA-CDC AR Bank	18.87
**Other *Candida* spp.**			
M175	*Candida albicans*	MCC, NYSDOH	0
M642	*C. blankii*	MCC, NYSDOH	0
M3193	*C. bracarensis*	MCC, NYSDOH	0
M5164	*C. ciferrii*	MCC, NYSDOH	0
M4363	*C. colliculosa*	MCC, NYSDOH	0
M314	*C. dubliniensis*	MCC, NYSDOH	0
M3051	*C. duobushaemulonii*	MCC, NYSDOH	0
M4625	*C. duobushaemulonii*	MCC, NYSDOH	0
M5690	*C. duobushaemulonii*	MCC, NYSDOH	0
M4831	*C. fabianii*	MCC, NYSDOH	0
M4572	*C. famata*	MCC, NYSDOH	0
M208	*C. glabrata*	MCC, NYSDOH	0
M446	*C. guillermondii*	MCC, NYSDOH	0
M5659	*C. haemulonii*	MCC, NYSDOH	0
M1081	*C. inconspicua*	MCC, NYSDOH	0
M4187	*C. intermedia*	MCC, NYSDOH	0
M3968	*C. kefyr*	MCC, NYSDOH	0
M3221	*C. krusei*	MCC, NYSDOH	0
M2840	*C. lipolytica*	MCC, NYSDOH	0
M240	*C. lusitaniae*	MCC, NYSDOH	0
M4430	*C. metapsilosis*	MCC, NYSDOH	0
M2717	*C. norvegensis*	MCC, NYSDOH	0

M4903	*C. orthopsilosis*	MCC, NYSDOH	0
M130	*C. parapsilosis*	MCC, NYSDOH	0
M4221	*C. propengiesseri*	MCC, NYSDOH	0
M3491	*C. quercitrusa*	MCC, NYSDOH	0
M3455	*C. rugosa*	MCC, NYSDOH	0
M3919	*C. sorbovorans*	MCC, NYSDOH	0
M15725	*C. zeylanoides*	MCC, NYSDOH	0
M1645	*Cryptococcus gattii* (NIH 444)	MCC, NYSDOH	0
M4758	*C. laurentii*	MCC, NYSDOH	0
M4909	*C. neoformans*	MCC, NYSDOH	0
M135	*C. neoformans* var. *grubii* (H99)	MCC, NYSDOH	0
M2383	*C. neoformans* var. *neoformans* (NIH12)	MCC, NYSDOH	0
M277	*Geotrichum capitatum*	MCC, NYSDOH	0
M133	*Kluyveromyces maxianus*	MCC, NYSDOH	0
M4920	*Rhodotorula mucilaginosa*	MCC, NYSDOH	0
M275	*R. rubrum*	MCC, NYSDOH	0
M78	*Saccharomyces cerevisiae*	MCC, NYSDOH	0
M205	*Trichosporon ashaii*	MCC, NYSDOH	0
**Mold species**			
M5499	*Aspergillus flavus*	MCC, NYSDOH	0
M5414	*A. fumigatus*	MCC, NYSDOH	0
M3319	*A. glaucus*	MCC, NYSDOH	0
M4737	*A. nidulans*	MCC, NYSDOH	0
M4355	*A. oryzae*	MCC, NYSDOH	0
M5370	*A. sydowii*	MCC, NYSDOH	0
M5321	*A. terreus*	MCC, NYSDOH	0
M4819	*A. versicolor*	MCC, NYSDOH	0
M4293	*Mucor circinelloides*	MCC, NYSDOH	0
**Bacterial spp.**			
DSM7312	*Aeromonas enteropelogenes*	BCC, NYSDOH	0
CDCBC3133	*Bacillus cereus*	BCC, NYSDOH	0
ATCC6051	*B. subtilis*	BCC, NYSDOH	0
ATCC33559	*Campylombacter coli*	BCC, NYSDOH	0
ATCC33291	*C. jejuni*	BCC, NYSDOH	0
NYS-08-6620	*Clostridium difficile*	BCC, NYSDOH	0
ATCC51434	*Escherichia coli 0157: H7*	BCC, NYSDOH	0
ATCC BAA-181	*E. coli STEC*	BCC, NYSDOH	0
ATCC19114	*Listeria monocytogenes*	BCC, NYSDOH	0
ATCC13076	*Salmonella enteritidis*	BCC, NYSDOH	0
ATCC14028	*S. typhimurium*	BCC, NYSDOH	0
ATCC12022	*Shigella flexneri*	BCC, NYSDOH	0
ATCC14458	*Staphylococcus aureus*	BCC, NYSDOH	0
ATCC17749	*Vibro alginolyticus*	BCC, NYSDOH	0
WC-33114	*Yersinia enterocolitica*	BCC, NYSDOH	0
**Parasite spp.**			
16-19165	*Babesia microti*	Parasitology, NYSDOH	0

16-19325	*Plasmodium falciparum*	Parasitology, NYSDOH	0
15-62007	*P. malariae*	Parasitology, NYSDOH	0
16-8664	*P. ovale*	Parasitology, NYSDOH	0
16-14201	*P. vivax*	Parasitology, NYSDOH	0
**Viral spp.**			
S-1	*Lentivirus HIV-2*	Virology, NYSDOH	0
S-2	*Orthoheppadnavirus hepatitis B virus*	Virology, NYSDOH	0

MCC, Mycology Culture Collection; BCC, Bacterial Culture Collection; NYSDOH, New York State Department of Health; FDA-CDC AR, Food & Drug Administration-Center for Diseases Control Antibiotic Resistant; ATCC, American Type Culture Collection

Supplementary Table 4. *Candida auris* detection in the spiked surveillance samples (swabs and sponges) by real-time PCR assay

Swabs	Level	CFU/50 µl	Yeast CFU/Rxn)	No. Samples Tested	Mean Ct ± SD	% CV
C. auris	High	10^5	10^4	10	20.51 ± 0.47	2.27
	Moderate	10^3	10^2	10	30.07 ± 0.35	1.15
	Low	10^2	10^1	10	33.52 ± 0.61	1.81
Candida spp.	Moderate	10^3	10^2	10	0.0 (Undet)	0
**Sponges**						
C. auris	High	10^5	10^4	10	22.08 ± 1.26	5.71
	Moderate	10^3	10^2	10	31.56 ± 0.60	1.91
	Low	10^2	10^1	10	35.51 ± 1.25	3.52
Candida spp.	Moderate	10^3	10^2	10	0.0 (Undet)	0

Supplementary Fig. 1. Multiple alignment of *ITS2* gene of *C. auris* and other closely related *Candida* species.

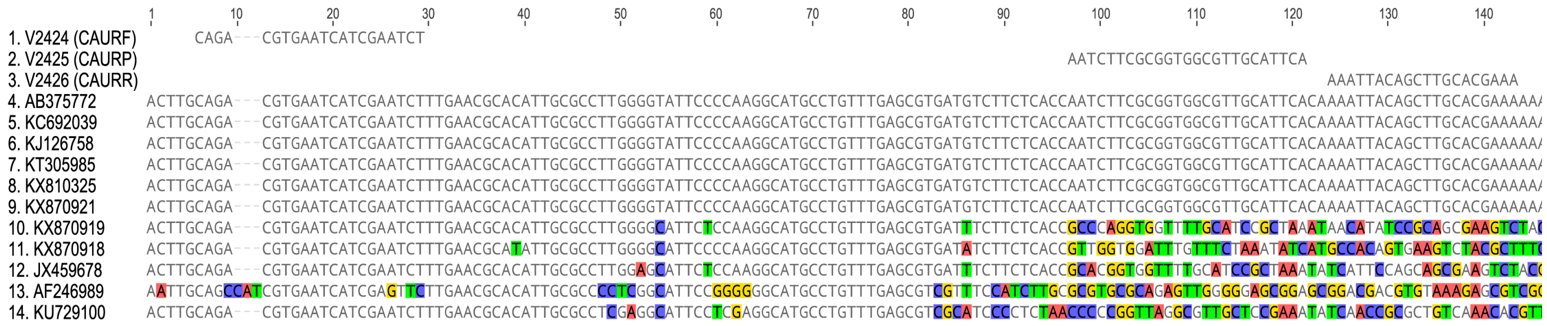

Supplementary Figure 2. Specificity assessment of *C. auris* real-time PCR assay in actual surveillance samples negative for *C. auris* DNA.

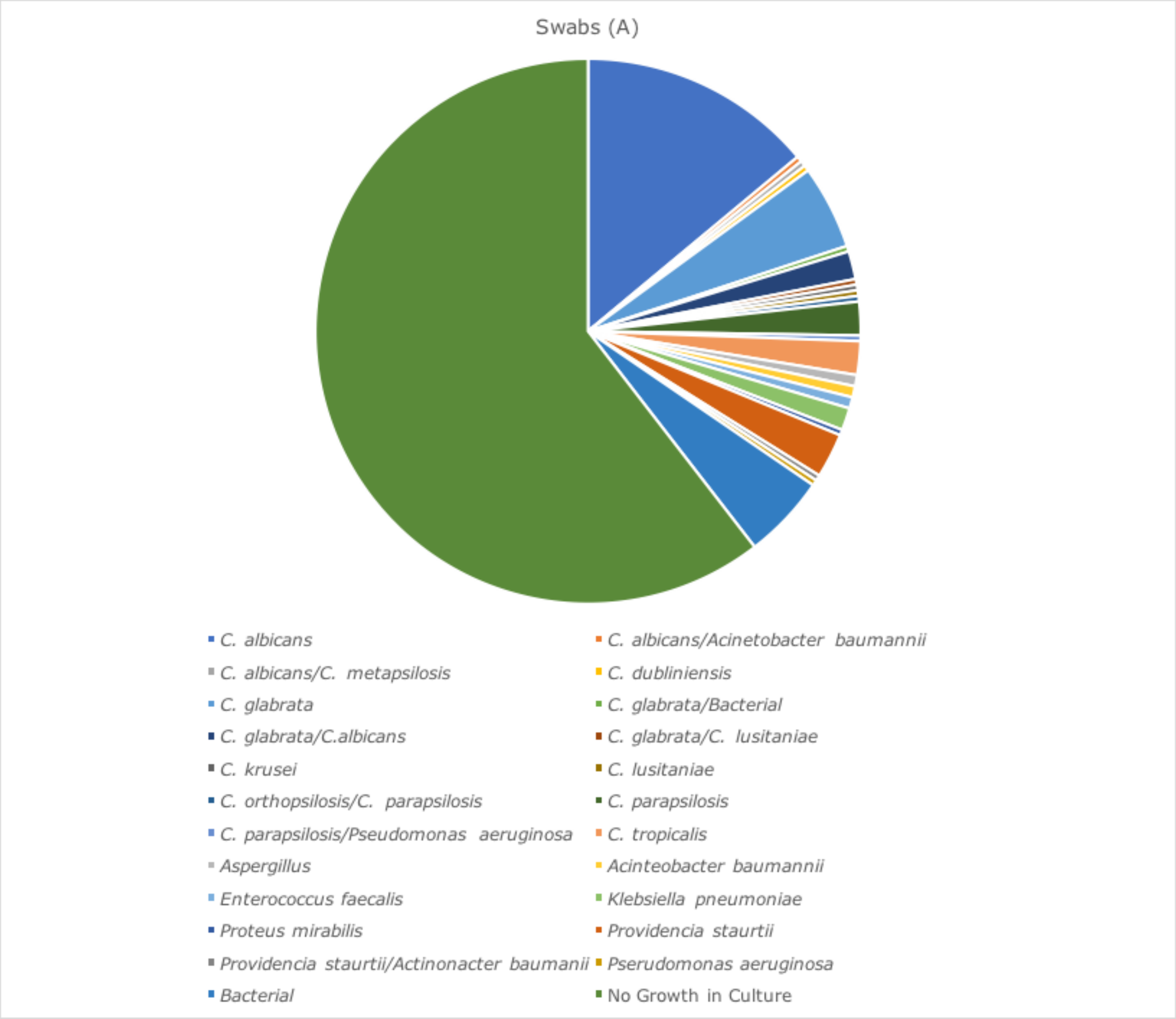

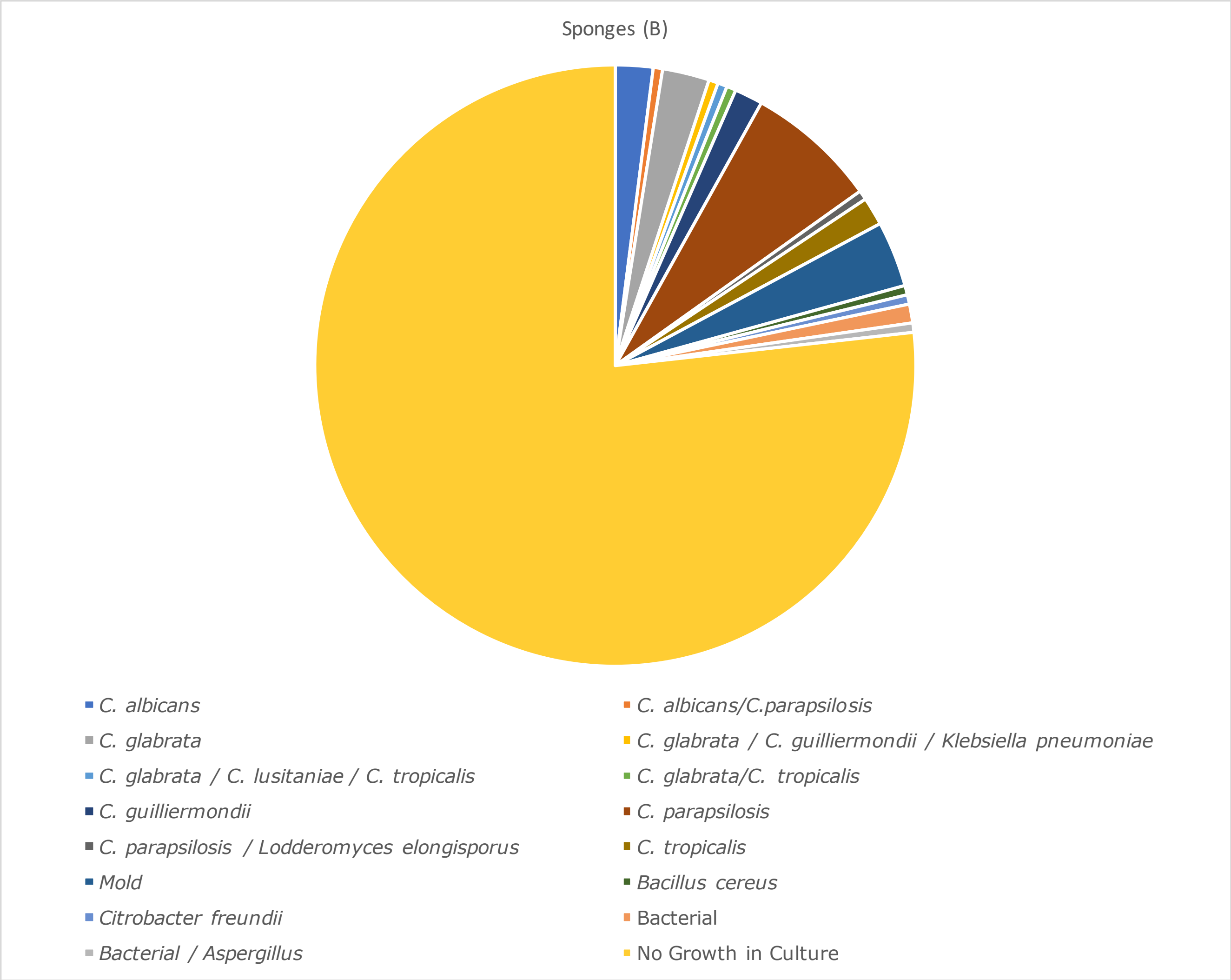
Sponges (B)
C. albicans
C. albicans/C.parapsilosis
C. glabrata
C. glabrata / C. guilliermondii / Klebsiella pneumoniae
C. glabrata / C. lusitaniae / C. tropicalis
C. glabrata/C. tropicalis
C. guilliermondii
C. parapsilosis
C. parapsilosis / Lodderomyces elongisporus
C. tropicalis
Mold
Bacillus cereus
Citrobacter freundii
Bacterial
Bacterial / Aspergillus
No Growth in Culture
