# Supplementary material for: Development and Validation of a Real-Time PCR Assay for Rapid Detection of Candida auris from Surveillance Samples
Source: J Clin Microbiol. 2018 Jan 24;56(2):e01223-17. doi: 10.1128/JCM.01223-17 (PMC5786737; doi:10.1128/JCM.01223-17)
Supplement: Supplemental material [file JCM.01223-17_zjm999095818s2.pdf]

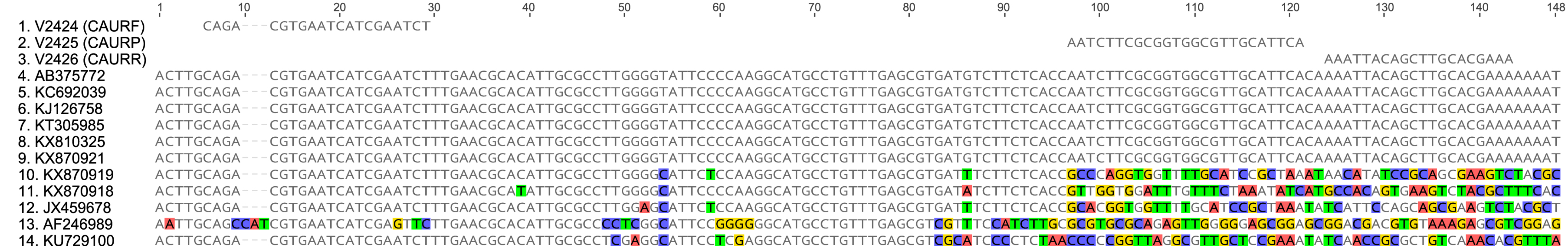

1 10 20 30 40 50 60 70 80 90 100 110 120 130 140 148
1. V2424 (CAURF)
CAGA---CGTGAATCATCGAATCT
2. V2425 (CAURP)
AATCTTCGCGGTGGCGTTGCATTCA
3. V2426 (CAURR)
AAATTACAGCTTGCACGAAA
4. AB375772
ACTTGCAGA---CGTGAATCATCGAATCTTTGAACGCACATTGCGCCTTGGGGTATTCCCCAAGGCATGCCTGTTTGAGCGTGATGTCTTCTCACCAATCTTCGCGGTGGCGTTGCATTCACAAAATTACAGCTTGCACGAAAAAAT
5. KC692039
ACTTGCAGA---CGTGAATCATCGAATCTTTGAACGCACATTGCGCCTTGGGGTATTCCCCAAGGCATGCCTGTTTGAGCGTGATGTCTTCTCACCAATCTTCGCGGTGGCGTTGCATTCACAAAATTACAGCTTGCACGAAAAAAT
6. KJ126758
ACTTGCAGA---CGTGAATCATCGAATCTTTGAACGCACATTGCGCCTTGGGGTATTCCCCAAGGCATGCCTGTTTGAGCGTGATGTCTTCTCACCAATCTTCGCGGTGGCGTTGCATTCACAAAATTACAGCTTGCACGAAAAAAT
7. KT305985
ACTTGCAGA---CGTGAATCATCGAATCTTTGAACGCACATTGCGCCTTGGGGTATTCCCCAAGGCATGCCTGTTTGAGCGTGATGTCTTCTCACCAATCTTCGCGGTGGCGTTGCATTCACAAAATTACAGCTTGCACGAAAAAAT
8. KX810325
ACTTGCAGA---CGTGAATCATCGAATCTTTGAACGCACATTGCGCCTTGGGGTATTCCCCAAGGCATGCCTGTTTGAGCGTGATGTCTTCTCACCAATCTTCGCGGTGGCGTTGCATTCACAAAATTACAGCTTGCACGAAAAAAT
9. KX870921
ACTTGCAGA---CGTGAATCATCGAATCTTTGAACGCACATTGCGCCTTGGGGTATTCCCCAAGGCATGCCTGTTTGAGCGTGATGTCTTCTCACCAATCTTCGCGGTGGCGTTGCATTCACAAAATTACAGCTTGCACGAAAAAAT
10. KX870919
11. KX870918
12. JX459678
13. AF246989
14. KU729100
