# Supplementary material for: Development and Validation of a Real-Time PCR Assay for Rapid Detection of Candida auris from Surveillance Samples
Source: J Clin Microbiol. 2018 Jan 24;56(2):e01223-17. doi: 10.1128/JCM.01223-17 (PMC5786737; doi:10.1128/JCM.01223-17)
Supplement: Supplemental material [file JCM.01223-17_zjm999095818s3.pdf]

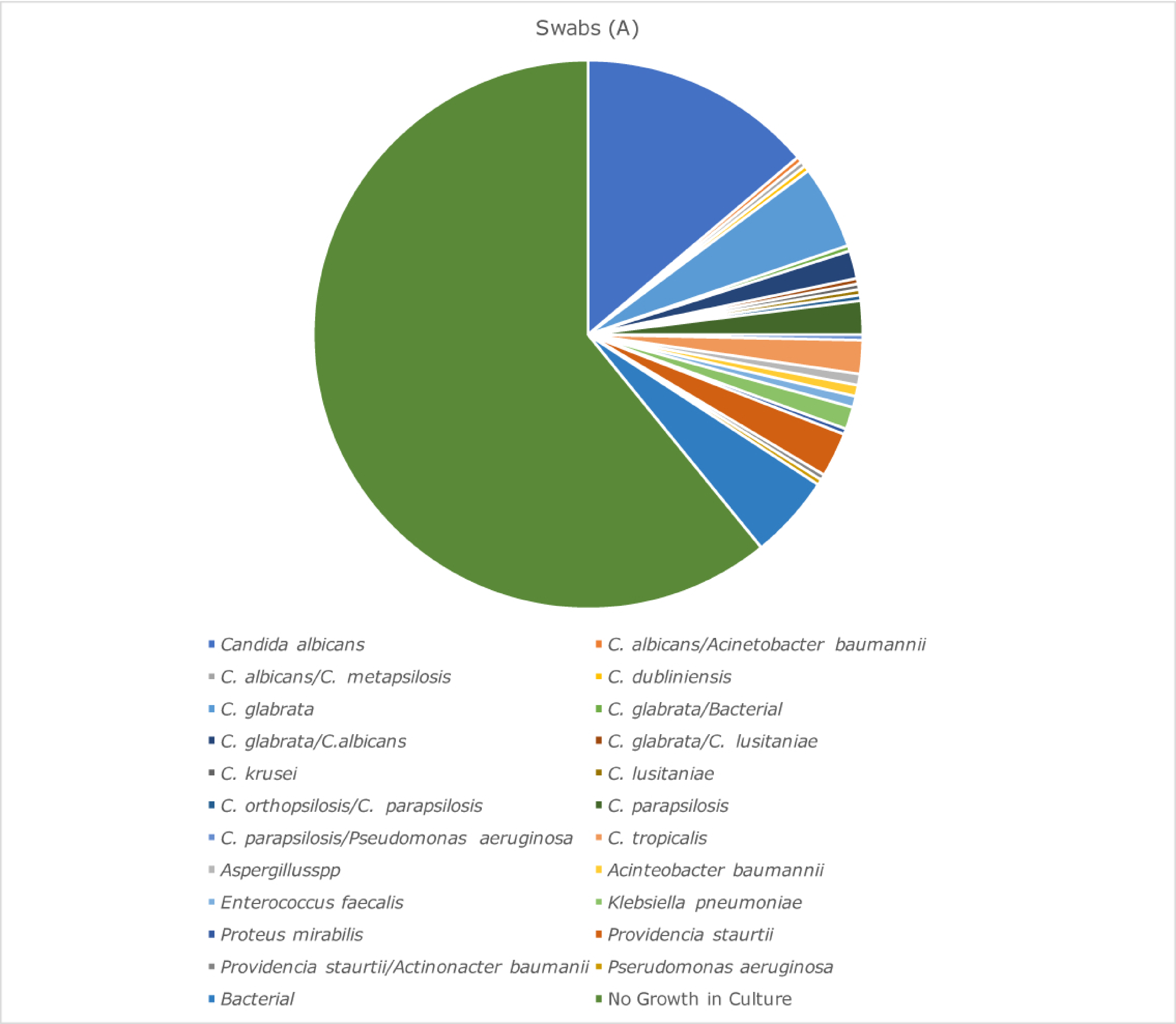
Swabs (A)
Candida albicans
C. albicans/Acinetobacter baumannii
C. albicans/C. metapsilosis
C. dubliniensis
C. glabrata
C. glabrata/Bacterial
C. glabrata/C.albicans
C. glabrata/C. lusitaniae
C. krusei
C. lusitaniae
C. orthopsilosis/C. parapsilosis
C. parapsilosis
C. parapsilosis/Pseudomonas aeruginosa
C. tropicalis
Aspergillusspp
Acinteobacter baumannii
Enterococcus faecalis
Klebsiella pneumoniae
Proteus mirabilis
Providencia staurtii
Providencia staurtii/Actinonacter baumanii
Pserudomonas aeruginosa
Bacterial
No Growth in Culture

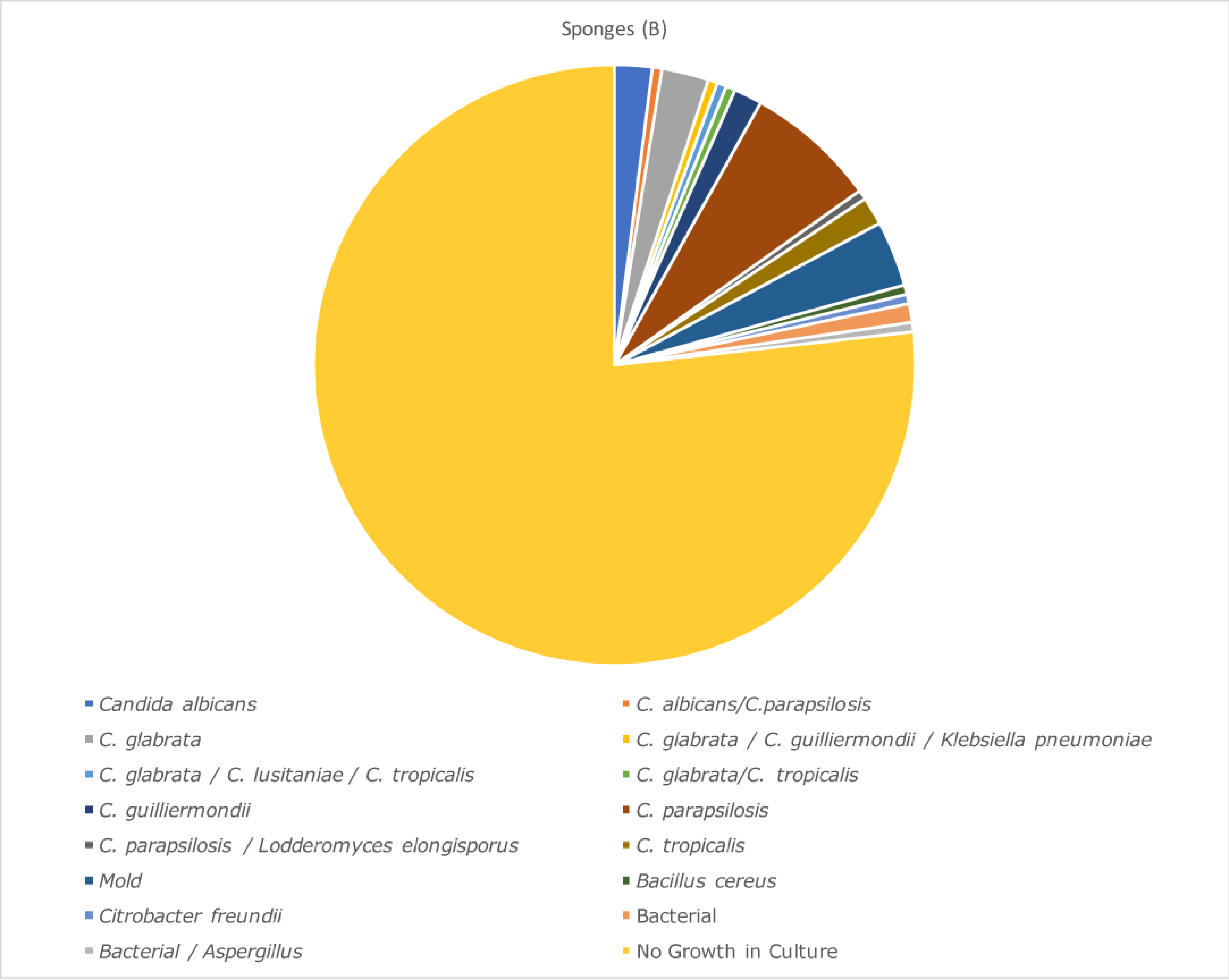
Sponges (B)
Candida albicans
C. albicans/C.parapsilosis
C. glabrata
C. glabrata / C. guilliermondii / Klebsiella pneumoniae
C. glabrata / C. lusitaniae / C. tropicalis
C. glabrata/C. tropicalis
C. guilliermondii
C. parapsilosis
C. parapsilosis / Lodderomyces elongisporus
C. tropicalis
Mold
Bacillus cereus
Citrobacter freundii
Bacterial
Bacterial / Aspergillus
No Growth in Culture
